# How Can Stress Be Controlled in Endodontically Treated Teeth? A 3D Finite Element Analysis

**DOI:** 10.1155/2013/426134

**Published:** 2013-07-15

**Authors:** İhsan Yıkılgan, Oya Bala

**Affiliations:** Department of Restorative Dentistry, Faculty of Dentistry, Gazi University, 06510 Emek, Ankara, Turkey

## Abstract

The aim of this study was to analyze the stresses that develop by oblique and vertical forces in endodontically treated maxillary second premolars that were restored with resin composite. Additionally, in our study the effects of the different restorative approaches and use of different base materials on stress formation were analyzed using three-dimensional finite element stress analysis. For restoration, the models representing both cusp capping, palatinal cusp capping, standard MOD restoration, and use of woven fiber in occlusal part were prepared. In all models, oblique forces caused more stress than did vertical forces. Materials with low elastic moduli cause high amounts of stress, whereas materials with elastic moduli similar to that of dental tissues cause low amounts of stress. Additional approaches such as cusp capping, functional cusp capping, and woven fiber use do not affect stress formation on the tooth after endodontic treatment.

## 1. Introduction

Many factors affect the success of endodontic treatment throughout its duration. Different approaches at various stages, such as the preparation technique, irrigation regimen, working length measurement, and obturation technique, have long-term functional effects on endodontically treated teeth. For clinical success, restoration after endodontic treatment is as important as the applications used during treatment. Many studies have suggested a direct connection between restoration quality and the success of endodontic treatment [1, 2].

Whereas healthy teeth may break because of traumatic factors [3], endodontically treated teeth are more fragile than vital teeth and can break because of typical functional factors. The main reason for such fracture is the loss of substance during caries elimination and the preparation of endodontic access cavities. Removing the marginal walls, especially in occlusal areas, during preparation negatively affects the fracture resistance of endodontically treated teeth [4]. Dehydration, collagen cruciate ligament loss, and dentin loss after endodontic treatment also negatively affect fracture resistance. 

Teeth that show little substance loss after endodontic treatment can be restored conservatively. Sound dentinal tissue with 1.5 mm thickness and 3-4 mm buccal and lingual heights is required to restore teeth conservatively [5]. Various materials, such as amalgam, composite resins, metal alloys, and dental ceramics, are used for conservative tooth restoration. Among these materials, composite resins are usually preferred for the direct restoration of teeth after endodontic treatment.

Various approaches are currently used to reduce or eliminate stress formation on dental tissues while restoring teeth that have been endodontically treated with composite resin. One approach is the use of base materials, such as resin-modified glass ionomer or fluid composite resin, under composite restorations. The use of base materials reportedly absorbs stress and thus can be beneficial although it remains controversial [6–8].

Composite restoration can be performed using different approaches to increase the fracture resistance of other dental tissues after endodontic treatment and to prevent the formation of undesired stress. One approach is cusp capping with composite resin, which is performed by slightly reducing cusp height and then covering the reduced cusps with composite resin. This procedure can be applied to functional or multiple cusps. Cusp capping reportedly decreases stress formation while increasing the fracture resistance of teeth after endodontic treatment [9–12].

Another restorative approach is the placement of woven fiber in the composite restoration to increase its mechanical strength [13, 14]. In studies evaluating the effect of woven fiber use on the fracture resistance of endodontically treated teeth, the fiber has been placed in the restoration or used to splint cusps [4, 13–18]. Although some studies have shown that the use of woven fiber increases the fracture resistance of teeth [13, 14, 19], others have found no significant effect [15–17].

In the previously studies, the effects of the use of base material and woven fiber on the stress formation in endodontically treated teeth have not been analyzed. The effect of cusp capping on the stress formation in endodontically treated teeth has been analyzed in a limited number of studies, but different types of cusp capping have not been compared. In addition, there is not a comprehensive study which can guide clinicians in the conservative restoration of endodontically treated teeth.

The aim of this study was to analyze (1) the different composite restoration types (cupping one cusp, cupping two cusps, using woven fiber) in endodontically treated maxillary second premolars, (2) the use of different base materials (fluid composite, resin-modified glass ionomer) under composite restorations, and (3) the effects of occlusal forces applied from different directions on stress in other dental tissues with a three-dimensional (3D) finite element stress analysis.

## 2. Materials and Methods

### 2.1. Master Model Preparation

**Figure 1:**
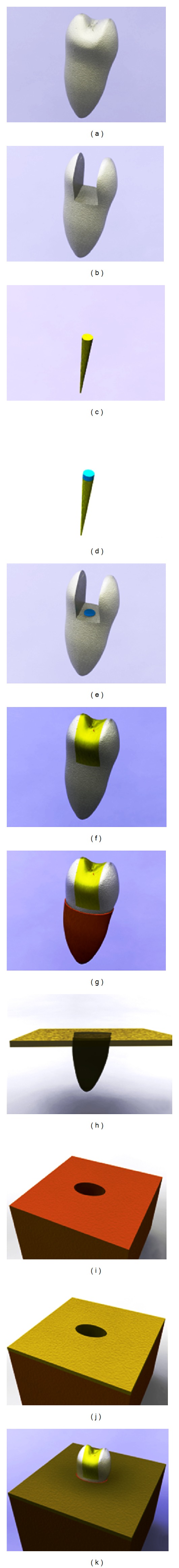
Model preparation, (a) sound tooth model, (b) MOD and endodontic access cavity, (c, d) root-canal filling and resin modified glass ionomer, (e) adaptation of root-canal filling to model, (f) restoration, (g) modeling of PDL, (h, i, j) lamina dura, cortical, and cancellous bone, and (k) model of the endodontically treated tooth.

A single-rooted, single-canal, endodontically treated maxillary second premolar with mesial and distal caries was evaluated in this study. First, images of the tooth were taken from different perspectives and in different dimensions using Wheeler's atlas. The images of the premolar were then scaled to create a model according to these dimensions using Rhinoceros software (ver. 4.0; McNeil Inc., NJ, USA) (Figure 1(a)). Mesio-occluso-distal (MOD) and endodontic access cavities were prepared on the tooth models, and root canal fillings were positioned (Figures 1(a), 1(b), 1(c), 1(d), and 1(e)). ProTaper F3 gutta percha (Dentsply Maillefer, Switzerland), which is designed for use with the F3 file of the ProTaper rotary instrument for root canal filling, was adapted to the root canal system by modeling to 1 mm short of the apex. Lamina dura (0.25 mm), periodontal ligament (0.25 mm), and cortical and cancellous bone (≥1.5 mm) were modeled around the tooth root and matched with the tooth using the same software (Figures 1(g), 1(h), 1(i), and 1(j)). Next, the master model of the endodontically treated tooth was made so that access was closed with 1 mm resin-modified glass ionomer cement. Changes were then made to mathematically analyze the distribution of any stress occurring with the use of different restorative approaches on this master pattern (Figure 1(k)).

### 2.2. Restorative Approaches

Models were made assuming the use of resin-modified ionomer cement and fluid composite resin as base materials. Four different approaches to maxillary tooth restoration were evaluated: capping two cusps, cupping the palatinal cusp, and using woven fiber on the occlusal area and standard MOD restoration. 

**Figure 2:**
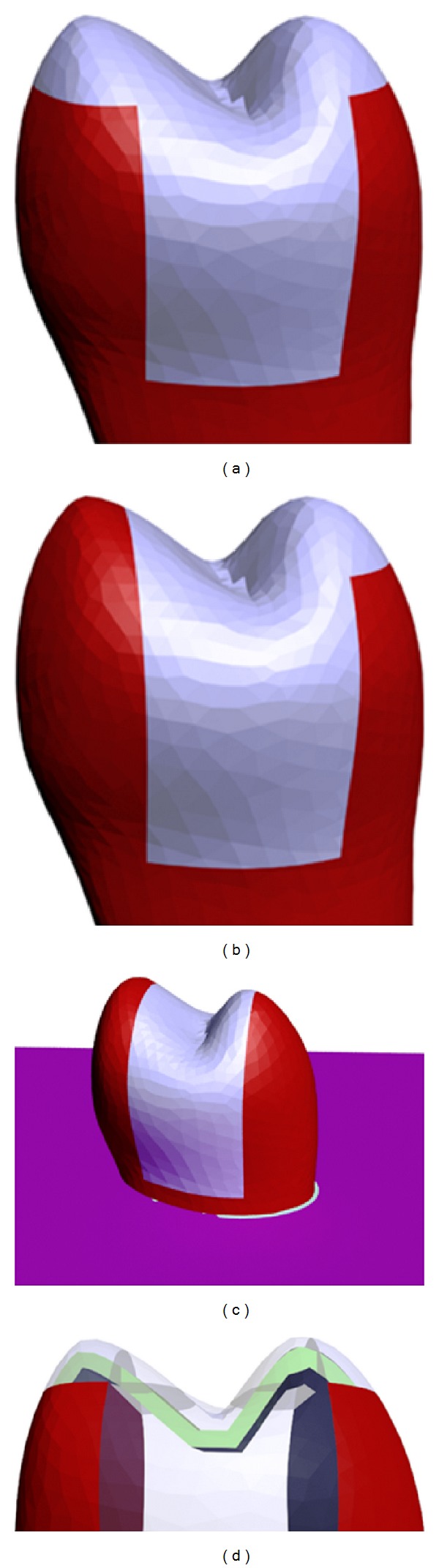
Restorative approaches (a) buccal + palatinal cusps capped, (b) palatinal cusps capped, (c) MOD restoration, and (d) woven fiber used.

Models that underwent capping of two cusps were prepared by simulating 2 mm vertical reductions of the buccal and palatinal cusps parallel to the long axis of the tooth on the master model. The obtained spaces were then filled with composite resin (Figure 2(a)).

Similarly, in models in which only the palatinal cusp was covered, the palatinal cusp was formed of composite resin and the buccal cusp of dental tissue (Figure 2(b)).

No additional restoration was performed with composite resin on models that underwent MOD restoration (Figure 2(c)).

For restorations in which woven fiber was used, a 3 mm wide and 2 mm deep space was modeled in the bucco-palatinal direction over the buccal and palatinal cusps on the master model. Woven fiber was then placed in the space so that it was consistent with the occlusal gradient of the tooth. Finally, the upper portion of the woven fiber was covered with composite resin (Figure 2(d)).

As a result, 12 models of 12 different restorative approaches were obtained.

Tooth models prepared with Rhinoceros 4.0 software were transferred to Algor Fempro software (ALGOR, Inc. 150 Beta Drive Pittsburgh, PA, USA) in STL format and meshed for analysis. For meshing, the models comprised 10-knot point (brick-type) units when possible. To obtain realistic results, we selected an excessive number of units for all dimensions of the tooth models within the bounds of the program. A total of 167,606 units and 30,172 knots were used in mathematical models that included scripts.

Young's modulus and Poisson's ratio, which describe the physical characteristics of each structure, were loaded into the software to identify the materials from which existing structures on the models prepared with Algor Fempro software were made (Table 1) [20, 21]. Solid features were accepted as linearly resilient, homogenous, and isotropic in the program.

### 2.3. Determination of Contact Surfaces on the Models

All components were assumed to be in contact with one another for the identification of surface relationships among the model's parts and analysis of the mathematical models.

### 2.4. Border Conditions

Zero motion and rotation were identified at six degrees of freedom from the side and upper surfaces of dental tissues.

### 2.5. Loading and Stress Analyses

Two different scripts were evaluated in the study. In the first script, a 100 N force was applied to the central fossae of restored tooth models parallel to the long axis of the tooth (vertical). In the second script, a 100 N force was applied to the palatinal cusps of the tooth models at a 45° angle to the long axis of the tooth (oblique).

Stresses arising from the restorative approach and script differences were evaluated with 3D finite element stress analysis, and Von Mises stress rates were recorded.

## 3. Results

Maximum stress values in models to which vertical and oblique forces were applied are shown in Table 2. In all models, enamel in the cervical area and at the cementoenamel junction showed the highest stress values after force application. 

When the models were evaluated according to restoration type, differences were observed between models to which vertical forces were applied and those to which oblique forces were applied. The highest stress values were observed in models in which woven fiber was used, compared with those to which vertical forces were applied. Whereas the stress values for other models were similar, those in which two cusps were covered yielded the lowest stress value. Specimens in which woven fiber was used showed lower stress values than did those to which oblique forces were applied. Whereas the stress values for other specimens were similar, those undergoing MOD restoration yielded the highest stress values.

Lower stress values were obtained in models with no base material than in those in which base material was used. Among models with base materials, lower stress values were obtained for resin-modified glass ionomer cement than for fluid composite.

**Figure 3:**
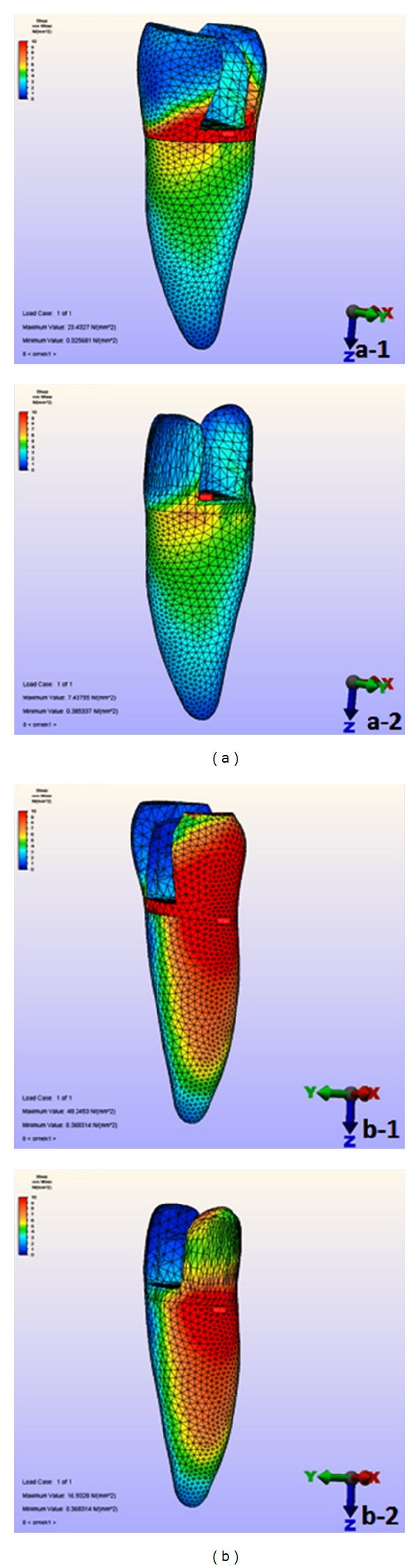
Principal stress distribution of Model 1 (buccal + palatinal cusps capped): (a) vertikal force applied ((a-1) enamel and dentin, (a-2) dentin) and (b) oblique force applied ((b-1) enamel and dentin, (b-2) dentin).

Higher stress values were obtained in models to which oblique forces were applied than in those to which vertical forces were applied (Figure 3).

## 4. Discussion

The effects of masticator forces on endodontically treated teeth have been evaluated by stress analysis and fracture resistance studies. Fracture resistance tests are important because they determine the maximum strength that teeth resist while functioning and enable the evaluation of tooth reaction to instant force application. However, such tests cannot evaluate fatigue in other dental tissues according to masticator forces. Thus, stress analysis methods are currently considered to be superior to fracture resistance tests because they provide comparative information about dental tissues, restoration fatigue, and deformation over time with the use of different restorative techniques.

Teeth are exposed to many forces from different parts of the mouth. Because the evaluation of all such forces was difficult on the 12 models prepared in our study, vertical and oblique forces, which are accepted as the basis of masticatory function, were evaluated. We found that oblique forces caused more stress than vertical forces in dental tissues because vertical forces applied to the teeth are transmitted to the root and, thereby, to periodontal tissues, causing less stress in the coronal tooth structures. However, oblique forces cause more stress in coronal than in periodontal tissues, resulting in more fatigue and injurious effects in coronal dental tissues. The results of previous studies support these findings [22, 23].

For both vertical and oblique force applications in our study, stress values were lower in specimens with no base models than in those in which the same restorative approaches were applied. The highest stress values were observed in models in which fluid composite resin was used as a base material, which may reflect differences in the elastic modulus of the materials. Our results are supported by studies showing that materials with elastic moduli similar to that of dental tissues transmit functional forces with less stress, whereas materials with low elastic moduli cause severe stress [5, 24]. Shubhashini et al. [24] evaluated the effects of composite resin, fluid composite resin, resin-modified glass ionomer cement, and glass ionomer cement on stress distribution in class V restorations using finite element stress analysis. They reported that the least stress was observed with composite resin, which has an elastic modulus similar to that of dental tissues. The authors concluded that materials with such elastic moduli may cause less stress.

The restoration types play an important role in the clinical prognosis of endodontically treated teeth. Fracture resistance of teeth is known to decrease after endodontic treatment, and the amount of stress in dental tissues increases in the face of functional forces. Many studies have revealed the ideal restorative approaches that increase fracture resistance of remaining dental tissues after endodontic treatment and enable minimal stress transmission [10, 18, 19, 25–27]. The use of woven fiber, fiber posts, and cusp capping was generally preferred in these studies to strengthen tooth structure. In our study, the effects of woven fiber and cusp capping on stress creation were evaluated.

Most studies evaluating the effect of cusp capping on the fracture resistance of endodontically treated teeth has MOD cavities in the maxillary premolars. These studies have produced inconsistent results; some have suggested that cusp capping increases fracture resistance [11, 12, 25, 28], whereas others have found no significant effect [9, 27, 29].

In our study, cusp capping techniques (one or two cusp capping) were not superior to other restoration techniques in terms of stress creation, and they even caused more stress in some specimens subjected to vertical and oblique force applications. In contrast to our results, Jiang et al. [10] reported that cusp capping created more tolerable stress in other dental tissues when teeth were treated endodontically. This difference may be because our study and that of Jiang et al. used different models for finite element analysis. In our study, cortical and cancellous bone and the periodontal ligament were modeled separately. Absence of the periodontal ligament in Jiang et al.'s model may have markedly affected the amount and dispersion of stress.

The use of woven fiber in the restoration of endodontically treated teeth is a new approach. Woven fiber is placed in the restoration or used for cusp splinting. Some studies have evaluated the effect of woven fiber on the fracture resistance of endodontically treated teeth [13, 15, 19, 30]. However, its effect on stress creation has not been evaluated.

Belli et al. [14] reported that fracture resistance values were higher in specimens in which woven fiber was placed in the occlusal parts of the restoration, and that fracture resistance of specimens in which woven fiber was placed at the base of the cavity was superior to that of specimens restored with only composite resin (no woven fiber). Sengun et al. [15] evaluated the effect of buccal and lingual cusp splinting with woven fiber on the fracture resistance of endodontically treated teeth. The use of woven fiber did not increase fracture resistance, but caused the creation of fractures that could be restored. Lia Mondelli et al. [12] showed that the fracture strength of specimens in which cusps were splinted with woven fiber was significantly superior to those in which woven fiber was placed at the cavity base or center of the restoration. In our study, cusps were splinted to each other by placing woven fiber in the space opened in palatinal and buccal cusps. However, the effect of the difference in woven fiber position on stress creation was not evaluated.

Among models with woven fiber, higher stress values were observed in those to which vertical force was applied than in those to which oblique force was applied. These results suggest that the use of woven fiber may be an effective option enabling the toleration of oblique forces, which cause more stress.

## 5. Conclusions

1. In all models, oblique forces caused more stress than did vertical forces.
2. Materials with low elastic moduli cause high amounts of stress, whereas materials with elastic moduli similar to that of dental tissues cause low amounts of stress. Thus, the use of materials with elastic moduli similar tissues to that of dental for restoration after endodontic treatment creates less stress in other dental tissues.
3. Additional restoration types such as cusp capping, functional cusp capping, and woven fiber use do not affect stress formation on the tooth after endodontic treatment.
4. The results of our study do not reflect the clinical situation because they were obtained using computed-generated mathematical models. Our findings with regard to the use of different restorative approaches in endodontically treated maxillary second premolars should be supported with clinical follow-up studies and further research.

## Figures and Tables

**Table 1:** Material properties (Young's modulus and Poisson's ratio).

Material	Young's modulus (GPa)	Poisson's ratio	Reference
Enamel	41	0.31	[21]
Dentin	18,6	0.31	[21]
Cortical bone	13,7	0.30	[21]
Cancellous bone	1,37	0.30	[21]
Periodontal ligament	0.0000689	0.45	[21]
Composite resin	12	0.30	[21]
Woven fiber			
Longitudinal (*X*)	46	0.39	[22]
Transverse (*Y*)	7	0.29	[22]
Transverse (*Z*)	7	0.29	[22]
Fluid composite resin	5,1	0.27	[21]
RMGI	10,6	0.30	[21]
Guta perka	0.14	0.45	[21]

**Table 2:** Maximum stress values in models to which vertical and oblique forces were applied.

	Restoration type	Base	Vertical force			Oblique force		
			Max.	Enamel	Dentin	Max.	Enamel	Dentin
Model 1	Buccal + palatinal cusps capping	RMGI	23,2576	23,2576	7,47318	48,1163	48,1163	16,8935
Model 2	Palatinal cusp capping	RMGI	23,2593	23,2593	7,46765	52,6151	52,6151	18,3603
Model 3	MOD restoration	RMGI	23,2844	23,2844	7,48487	52,6108	52,6108	18,3592
Model 4	Woven fiber	RMGI	24,279	24,279	7,41309	52,7324	52,7324	18,3862
Model 5	Buccal + palatinal cusps capping	Fluid composite	25,019	25,019	7,99823	49,32	49,32	17,2984
Model 6	Palatinal cusp capping	Fluid composite	25,0536	25,0536	7,99245	49,3576	49,3576	17,3073
Model 7	MOD restoration	Fluid composite	25,078	25,078	8,01069	53,8333	53,8333	18,7693
Model 8	Woven fiber	Fluid composite	26,1037	26,1037	7,94491	53,9383	53,9383	18,7906
Model 9	Buccal + palatinal cusps capping	No base	22,0818	22,0818	7,15808	51,6907	51,6907	18,0553
Model 10	Palatinal cusp capping	No base	22,083	22,083	7,15272	48,4006	48,4006	16,6957
Model 11	MOD restoration	No base	22,1082	22,1082	7,16925	51,7573	51,7573	18,0709
Model 12	Woven fiber	No base	23,2833	23,0794	7,09289	44,0467	44,0467	15,1938
